# A Retrospective Assessment of Sputum Samples and Antimicrobial Resistance in COVID-19 Patients

**DOI:** 10.3390/pathogens12040620

**Published:** 2023-04-19

**Authors:** Talida Georgiana Cut, Adelina Mavrea, Alin Adrian Cumpanas, Dorin Novacescu, Cristian Iulian Oancea, Felix Bratosin, Adelina Raluca Marinescu, Ruxandra Laza, Alexandra Mocanu, Alexandru Silvius Pescariu, Diana Manolescu, Raluca Dumache, Alexandra Enache, Elena Hogea, Voichita Elena Lazureanu

**Affiliations:** 1 Department XIII, Discipline of Infectious Diseases, Victor Babes University of Medicine and Pharmacy Timisoara, E. Murgu Square, Nr. 2, 300041 Timisoara, Romania; 2 Doctoral School, Victor Babes University of Medicine and Pharmacy Timisoara, E. Murgu Square, Nr. 2, 300041 Timisoara, Romania; 3 Center for Ethics in Human Genetic Identifications, Victor Babes University of Medicine and Pharmacy Timisoara, E. Murgu Square, Nr. 2, 300041 Timisoara, Romania; 4 Academy of Romanian Scientists, Splaiul Independentei, Nr. 54, 50085 Bucharest, Romania; 5 Department VII, Internal Medicine II, Discipline of Cardiology, Victor Babes University of Medicine and Pharmacy Timisoara, E. Murgu Square, Nr. 2, 300041 Timisoara, Romania; 6 Department XV, Discipline of Urology, Victor Babes University of Medicine and Pharmacy Timisoara, E. Murgu Square, Nr. 2, 300041 Timisoara, Romania; 7 Department XIII, Discipline of Pneumology, Victor Babes University of Medicine and Pharmacy Timisoara, E. Murgu Square, Nr. 2, 300041 Timisoara, Romania; 8 Center for Research and Innovation in Precision Medicine of Respiratory Diseases (CRIPMRD), Victor Babes University of Medicine and Pharmacy Timisoara, E. Murgu Square, Nr. 2, 300041 Timisoara, Romania; 9 Department XV, Discipline of Radiology, Victor Babes University of Medicine and Pharmacy Timisoara, E. Murgu Square, Nr. 2, 300041 Timisoara, Romania; 10 Department VIII, Discipline of Forensic Medicine, Victor Babes University of Medicine and Pharmacy Timisoara, E. Murgu Square, Nr. 2, 300041 Timisoara, Romania; 11 Department XIV, Discipline of Microbiology, Victor Babes University of Medicine and Pharmacy Timisoara, E. Murgu Square, Nr. 2, 300041 Timisoara, Romania

**Keywords:** SARS-CoV-2, co-infection, superinfection, multidrug resistance, outcome

## Abstract

Data on bacterial or fungal pathogens and their impact on the mortality rates of Western Romanian COVID-19 patients are scarce. As a result, the purpose of this research was to determine the prevalence of bacterial and fungal co- and superinfections in Western Romanian adults with COVID-19, hospitalized in in-ward settings during the second half of the pandemic, and its distribution according to sociodemographic and clinical conditions. The unicentric retrospective observational study was conducted on 407 eligible patients. Expectorate sputum was selected as the sampling technique followed by routine microbiological investigations. A total of 31.5% of samples tested positive for *Pseudomonas aeruginosa*, followed by 26.2% having co-infections with *Klebsiella pneumoniae* among patients admitted with COVID-19. The third most common *Pathogenic bacteria* identified in the sputum samples was *Escherichia coli*, followed by *Acinetobacter baumannii* in 9.3% of samples. Commensal human pathogens caused respiratory infections in 67 patients, the most prevalent being *Streptococcus penumoniae*, followed by methicillin-sensitive and methicillin-resistant *Staphylococcus aureus*. A total of 53.4% of sputum samples tested positive for *Candida* spp., followed by 41.1% of samples with *Aspergillus* spp. growth. The three groups with positive microbial growth on sputum cultures had an equally proportional distribution of patients admitted to the ICU, with an average of 30%, compared with only 17.3% among hospitalized COVID-19 patients with negative sputum cultures (*p* = 0.003). More than 80% of all positive samples showed multidrug resistance. The high prevalence of bacterial and fungal co-infections and superinfections in COVID-19 patients mandates for strict and effective antimicrobial stewardship and infection control policies.

## 1. Introduction

The coronavirus disease 2019 (COVID-19), a zoonotic infection caused by severe acute respiratory syndrome coronavirus 2 (SARS-CoV-2), has swiftly emerged as the most severe pandemic of the last decades [1]. Along with raising major public health concerns, the infection brought an increase in inappropriate antimicrobial consumption and bacterial resistance [2,3,4,5,6]. A meta-analysis on 362,976 patients, by Langford et al., published in 2023, concluded that 60.8% of the bacterial infections in COVID-19 patients were resistant to antimicrobials [7].

In moderate-income countries such as Romania, where there is a well-established high burden of multidrug-resistant organisms in ICU and in-ward settings, superinfections in COVID-19 patients, especially with bacteria and fungi, are increasing the difficulties in diagnosis, management, and prognosis. In a study by Marinescu et al. on *Clostridioides difficile* infection (CDI) and COVID-19 in the Western Romanian population, 75% of the study group patients were treated with broad-spectrum antibiotics prior to hospital admission and 5% of patients with COVID-19 and CDI died of septic shock [8]. The most common antibiotic prescribed for outpatient treatment of COVID-19 in Romania was azithromycin, despite evidence against its use in these situations [9,10]. In the same tertiary unit, Laza et al. observed that secondary infections and sepsis contribute significantly to worsen the prognosis in terms of morbidity and mortality [11].

Although bacterial infections are known complications in viral respiratory infections, the radiological features are not completely specific, as both bacterial and viral pneumonia may generate consolidated foci [12,13,14]. Furthermore, SARS-CoV-2 can trigger a hyperinflammatory syndrome that resembles bacterial sepsis, with multi-organ failure and elevated inflammatory biomarkers [15,16,17,18,19,20]. The impairment in immune function contributes to increased susceptibility to bacterial or fungal co-infections and superinfections; however, the prevalence remains controversial [21].

The current study aims to further our overarching research goal of documenting the clinical and microbiological impact of the initially non-specific, and mostly symptomatic, COVID-19 clinical management strategies responsible for a massive spike in empirical and/or prophylactic prescription of antibiotics.

## 2. Materials and Methods

### 2.1. Study Design and Ethical Considerations

A retrospective statistical analysis of clinical and microbiological data, focusing on a later cohort of consecutive COVID-19 cases hospitalized within the second half of the pandemic, with concomitant respiratory co-infections and/or secondary superinfections, as confirmed by positive sputum cultures, was performed. We report on the emerging prevalence rates for the dominant respiratory pathogens and subspecies involved, as well as their associated antibiotic resistance profiles. Patients included in the study were admitted within the non-critical ward of the “Victor Babes” Infectious Diseases and Pneumophtisiology Hospital, located in Timisoara, the largest unit of its kind in Western Romania.

### 2.2. Inclusion Criteria and Study Variables

We examined all available case files for non-critical COVID-19 admissions within a six-month interval, i.e., between 1 July 2021 and 31 December 2021, and developed our current study population. As defined by our pre-established study inclusion criteria, the cohort included patients over 18 years old, with a positive molecular RT-PCR SARS-CoV-2 test from nasopharyngeal swabs and radiological findings suggestive of COVID-19. Upon initial evaluation, the presence of at least one of the following risk factors justified hospitalization: age 60 years or older, cardiovascular disease, chronic obstructive pulmonary disease, asthma, emphysema, diabetes, malignancy, obesity (BMI ≥ 30 kg/m^2^), chronic kidney disease, immunocompromised state (solid organ transplantation, recipient of immunosuppressive therapy, HIV/AIDS), pregnancy, sickle cell disease, dyspnea or increased respiratory rate (≥30 breaths per min), oxygen saturation ≤ 94% on room air or decrease in saturation to <90%, lymphopenia < 1000/mm^3^, D-dimer > 250 ng/mL, and CRP > 10 mg/L.

A total of 407 patients were included in the study for evaluation after satisfying the inclusion criteria. All patients documented to have been taking antibiotics before sputum sampling were excluded from the current analysis. Furthermore, when sputum sample cultures revealed a mixed co-infection, with multiple distinct infectious agents and/or subspecies, the case was excluded due to fears of sample cross-contamination.

Moving forward, for all the cases which met our study inclusion criteria, complete data sets were collected, focusing on the variables considered relevant for further statistical processing: (1) background (patient age group, patient’s sex, area of residence, body mass index distribution, smoking status, history of pre-existing lung disease, and number of comorbidities), (2) hospital admission and testing results (days from symptom onset until hospitalization, days from positive COVID-19 PCR test until hospitalization, hospitalization prior to COVID-19 infection, time of sputum sampling, multidrug resistance, and number of pathogens identified), (3) pathogen identification (*Pathogenic bacteria*, commensal flora, and fungal infection), (4) distribution of antimicrobial resistance, (5) biological findings (red blood cell count, platelet count, white blood cell count, lymphocyte count, hemoglobin levels, hematocrit, creatinine, blood urea nitrogen, glomerular filtration rate, fasting glucose, alanine aminotransferase, aspartate aminotransferase, international normalized ratio, ferritin, lactate dehydrogenase, lactate levels, C-reactive protein, interleukin-6, erythrocyte sedimentation rate, fibrinogen, and D-dimers), and (6) patient outcomes (severe complications, COVID-19 severity, oxygen saturation at admission, oxygen supplementation at admission, oxygen flow rate, intensive care unit (ICU) admission, duration of ICU stay, mortality, days from admission until death, and days of hospitalization).

### 2.3. Study Terminology and Sputum Sampling

1. Infections diagnosed within 48 h of hospital admission were classified as co-infections. Infections identified after 48 h of admission were classified as superinfections [4,22,23,24]. Biological samples for the diagnosis of co-infection/superinfection in COVID-19 patients were collected after evaluating the following clinical criteria: purulent sputum, persistent fever (>38 °C), deterioration of ventilatory parameters, or hemodynamic instability.
2. Laboratory criteria: worsening of leukocytosis or leucopenia, increased procalcitonin, or C-reactive protein.
3. Radiological criteria: progression/worsening of the chest radiological pattern, or onset of a pattern characteristic for bacterial pneumonia such as basal consolidation, nodules, cavitation, or pleural effusion.

Expectorate sputum was selected as the sampling technique and routine microbiological investigations were conducted at the medical microbiology laboratory using standard bacteriology. All isolates were first identified using the VITEK^®^ 2 GN and VITEK^®^ 2 GP ID cards (BioMérieux, Marcy, l’Etoile, France). Antimicrobial susceptibility tests were performed using the VITEK 2 GN AST-N222 and VITEK 2 AST GP 67 cards (BioMérieux, Marcy, l’Etoile, France).

### 2.4. Statistical Analysis

Data analysis was conducted using IBM SPSS Statistics v27 software (SPSS Inc., Chicago, IL, USA). The absolute (n) and relative (%) frequencies of categorical variables were calculated. Their proportions were tested using Chi-square and Fisher’s exact test. The available data were tested for normality with the Shapiro–Wilk test. We used the Mann–Whitney test to compare non-Gaussian variables and reported them by the median and interquartile range (IQR). The mean and standard deviation of continuous variables with a normal distribution were compared using the Student’s *t*-test (unpaired, independent samples) [14,21,25]. The threshold for statistical significance was 0.05. Kaplan–Meier curves were constructed for analyzing survival data. We adjusted the Kaplan–Meier survival curves for confounding factors using Cox proportional hazards regression analysis.

## 3. Results

### 3.1. Characteristics of the Study Cohort

Data collection identified 407 eligible patients that were hospitalized for COVID-19. There were 46 individuals with a positive sputum culture growing *Pathogenic bacteria*, 67 patients with positive sputum cultures for respiratory tissue-associated commensal bacteria, 51 cases identified with fungal growth in their sputum cultures, and 243 patients with negative sputum cultures. The mean age of patients with *Pathogenic bacteria* was 62.1 years (±12.0); the mean age of patients with positive sputum samples with commensal human pathogens was 66.8 (±13.7); the mean age of patients with positive fungal samples was 64.4 (±11.3), while the mean age of patients in the control group was 67.5 (±14.8), with no significant differences between groups (*p* > 0.05). It was observed that the majority of patients were elderly people over the age of 65, of which more than 52% were men. However, there were no statistically significant findings between the four comparison groups regarding their background (*p* > 0.05). The body mass index of the studied groups was, in approximately half of the cases analyzed, within the normal range, between 18.5 and 25.0 kg/m^2^. However, the proportion of COVID-19 patients with respiratory fungal infections was significantly different compared to the other groups, since underweight and/or overweight patients comprised more than 56% of all cases. Additionally, pre-existing lung disease was identified in approximately 14% of all patients included in the study, with a higher proportion among patients with fungal infections (17.6%). Other comorbidities were very common among the four study groups, with less than 5% of all patients having no pre-existing comorbid condition, as described in Table 1.

Table 2 describes testing results and hospital admission features of COVID-19 patients stratified by sputum culture results. It was observed that patients with fungal infections had a significantly shorter average duration from symptom onset until hospitalization (4.7 days), compared with 6.1 days in patients with *Pathogenic bacteria* identified in the sputum samples, 5.9 days among those with commensal flora infections, and 6.8 days in the control group (*p* < 0.001). However, the time elapsed from the first positive COVID-19 PCR test until hospitalization was approximately 4 days, without significant differences between groups (*p* > 0.05). Most sputum samples were taken within 48 h from hospital admission, and more than 80% of all samples showed multidrug resistance.

The distribution of antimicrobial resistance among COVID-19 patients stratified by categories of sputum samples presented in Table 3 identified only a low proportion of samples to be sensitive to all specific antimicrobial drugs, although the distribution proportion was not statistically significant between groups (*p* > 0.05). However, we observed a significantly higher proportion of antifungal-sensitive sputum samples (19.6%), compared with only 8.7% for sensitive samples among cultures positive for *Pathogenic bacteria* (*p* = 0.031). Most of the samples were resistant to two or three antimicrobials: 21.7% and 23.9% among the sputum samples positive for *Pathogenic bacteria*, respectively; 26.9% and 14.9% among the sputum samples positive for commensal pathogenic growth, respectively; and 25.5% and 27.5% of sputum samples were resistant for two and three antifungals, respectively. Still, 6.5% of samples and 4.5% of samples in the *Pathogenic bacteria* and commensal flora groups, respectively, were resistant to more than five antimicrobials.

### 3.2. Microbial Identification

**Figure 1:**
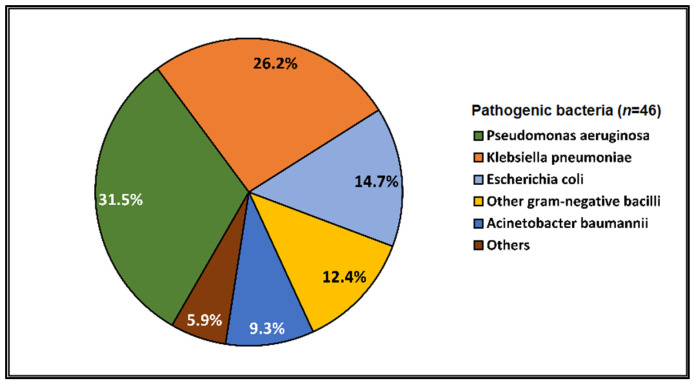
Distribution of *Pathogenic bacteria* identified in the sputum samples of hospitalized COVID-19 patients.

**Figure 2:**
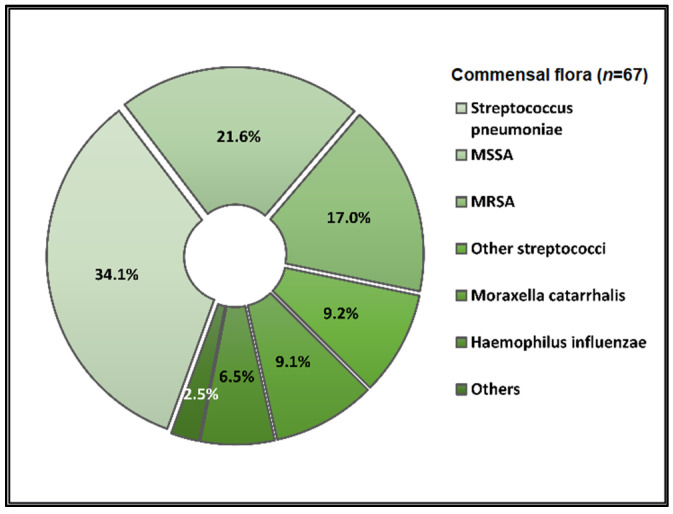
Distribution of commensal human pathogens identified in the sputum samples of hospitalized COVID-19 patients.

**Figure 3:**
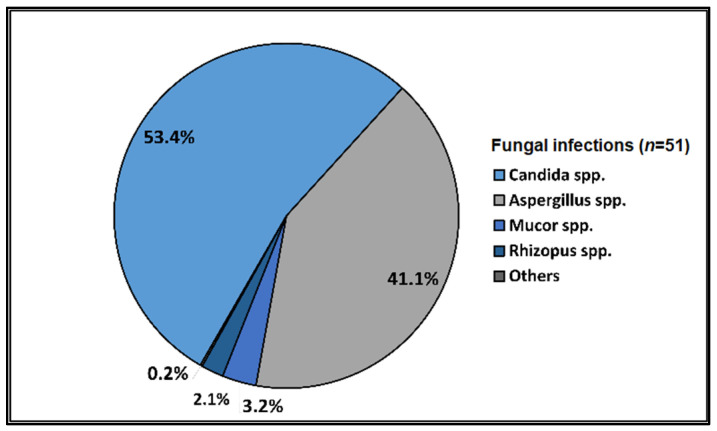
Distribution of fungi identified in the sputum samples of hospitalized COVID-19 patients.

The parallel pathogen identification among sputum samples was classified by *Pathogenic bacteria* for the respiratory tract, commensal human pathogens of the respiratory tract, and fungal infections of the respiratory tract, as described in Figure 1, Figure 2, and Figure 3, respectively. In total, 31.5% of samples were positive for *Pseudomonas aeruginosa*, followed by 26.2% with co-infections with *Klebsiella pneumoniae*, among patients admitted with COVID-19. The third most common *Pathogenic bacteria* identified in the sputum samples was *Escherichia coli*, followed by other Gram-negative bacilli, and *Acinetobacter baumannii* in 9.3% of samples. Commensal human pathogens caused respiratory infections in 67 patients with COVID-19, the most prevalent being *Streptococcus penumoniae* in 34.1% of patients, followed by methicillin-sensitive (21.6%) and methicillin-resistant *Staphylococcus aureus* (17.0%). The remaining sputum samples were confirmed for *Moraxella catarrhalis* in 9.1% of the samples and *Haemophilus influenzae* in 6.5%. Lastly, fungal infections among non-critical COVID-19 patients admitted to the infectious disease department identified a majority of 53.4% of samples positive for *Candida* spp. growth, followed by 41.1% of samples with *Aspergillus* spp. growth.

### 3.3. Biological Findings

The comparison of biological parameters between the study groups, as described in Table 4, found that COVID-19 patients with commensal flora respiratory co-infections had a significantly higher proportion of patients with elevated WBC, as compared with patients with fungal co-infections and the control group (85.1% vs. 56.1% vs. 46.9%, *p* < 0.001). On the contrary, patients with fungal co-infections had significantly more elevated lymphocyte counts (*p* = 0.002) and transaminase levels as compared to the control group of COVID-19 patients with negative sputum samples (25.5% vs. 10.7%, *p* = 0.041). Inflammatory markers were also statistically significantly different among the four study groups. High procalcitonin levels were observed among patients with *Pathogenic bacteria* and commensal flora respiratory co-infections (60.9% and 61.2% of samples outside the normal range, respectively), while IL-6, ESR, and CRP were equally elevated in the three groups with positive samples compared with the control group (*p* < 0.05).

### 3.4. Outcomes and Predictions

Table 5 describes the outcomes of COVID-19 patients stratified by sputum culture results. Patients with fungal co-infections had more severe complications (39.2% vs. 22.6% in the control group of negative samples, *p* = 0.028). Although the proportion of complications was different, the severity of SARS-CoV-2 infection at admission did not differ significantly between the study groups. However, patients with fungal co-infections had a significantly higher proportion of patients with oxygen saturations at admission lower than 92% (64.7% vs. 38.3% in the control group, *p* = 0.040). The three groups with positive microbial growth on sputum cultures had an equally proportional distribution of patients admitted to the ICU, with an average of 30% of them being admitted, compared with only 17.3% among hospitalized COVID-19 patients with negative sputum cultures (*p* = 0.003). Consequently, the duration of hospitalization, ICU stay, and mortality was much higher in these three groups than in the patients with negative samples.

**Figure 4:**
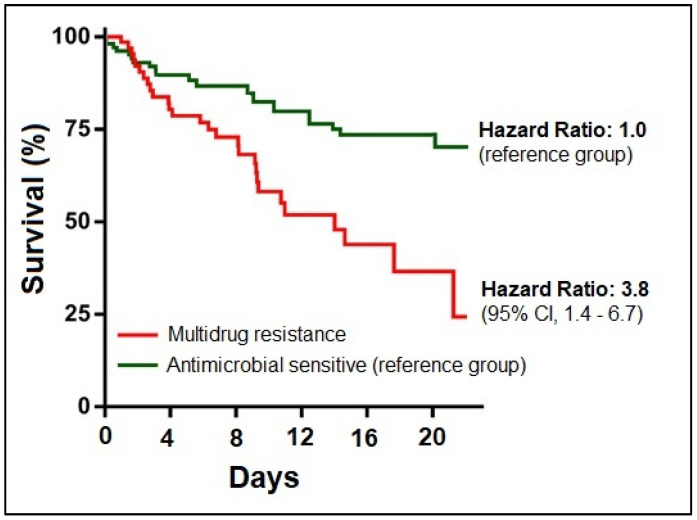
Kaplan–Meier probability curve of patients’ survival stratified by antimicrobial resistance status.

**Figure 5:**
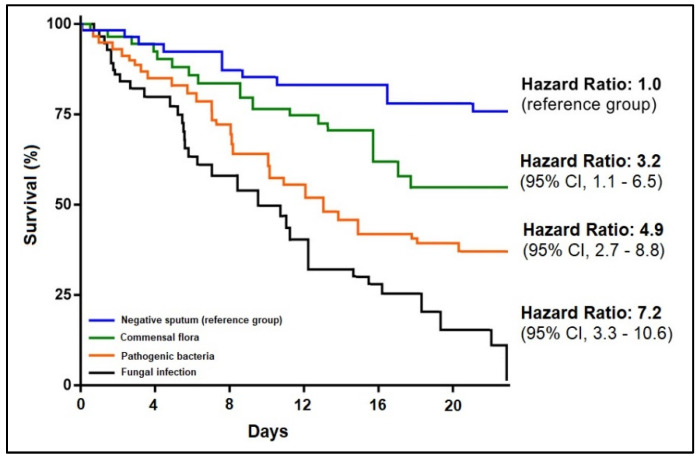
Kaplan–Meier probability curve of patients’ survival stratified by sputum culture results.

A Kaplan–Meier survival curve is presented in both Figure 4 and Figure 5, indicating a hazard ratio of 3.8 in hospitalized COVID-19 patients with bacterial and fungal respiratory co-infections that were multidrug resistant (95% CI = 1.4–6.7). Similarly, the survival hazard ratio was significantly different between the four study groups, with an HR of 3.2 among COVID-19 patients with respiratory tract commensal pathogens co-infections, an HR of 4.9 among those with *Pathogenic bacteria* respiratory co-infections, followed by the highest risk among those with fungal co-infections (HR = 7.2).

## 4. Discussion

A viral infection can destroy the respiratory tract of individuals upon viral spread [26]. Depending on the type of the virus, the functional changes include cell apoptosis, decreased mucosal clearance, reduced oxygen exchange, and impaired surfactant secretion [27].

SARS-CoV-2 can facilitate the colonization and attachment of bacteria to the host respiratory tissues, leading to mixed infections in connection with tissue destruction caused by this virus [27]. Defining the etiology of pneumonia with conventional diagnostic tests in these particular patients remains challenging as some experts argue that sputum cultures have low specificity and sensitivity [28,29,30,31,32].

Data available on secondary infections and antibiotic use in COVID-19 patients admitted to Western Romanian hospitals are limited. Furthermore, sputum cultures are cost efficient and have allowed us to assess the regional respiratory pathogens associated with COVID-19 pneumonia. In the framework of this study, we found rather high rates of bacterial co-infections and secondary infections (23.3% and 16.95%, respectively) compared to a study conducted by Timpau et al., where the reported figures were significantly lower (1.4% and 6.8%, respectively), but similar to the prevalence rates observed by Langfort et al., Novacescu et al., and Contou et al. [33,34,35,36]. Different studies have found a highly variable prevalence of bacterial superinfections in COVID-19 patients, ranging between 1% and 50%, which can be explained by differences in criteria and diagnostic tests [27,37,38,39,40]. Dubourg et al. performed a correlation study of the cultured bacteria from paired sputum and bronchoalveolar lavage fluid. The results suggested that the culture of sputum specimens may result in useful microbiologic diagnosis [41]. Furthermore, in 2021, Mazloomirad et al. published a study on hospital-acquired pneumonia in southwestern Iran and found no significant differences between bacteria isolated by either the culture or PCR methods [42].

Among the sociodemographic and clinical factors associated with positive sputum culture results, a significant relationship was found with males older than 65 years of age, which is very similar to that reported by Ripa et al. and Lv et al. [43,44,45].

In line with previous studies, *Pseudomonas aeruginosa* emerged as the most common *Pathogenic bacteria* identified in the sputum samples, followed by *Klebsiella pneumoniae* and *Escherichia coli* [29,31,46,47,48]. Compared to a meta-analysis study conducted by Musuuza et al., where the most frequent bacteria identified in superinfected patients was *Acinetobacter* spp., we found a rather reduced rate of 22% and 9.3%, respectively [43]. This can be explained by the fact that mechanical ventilation is frequently required by severe COVID-19 patients admitted in ICUs along with heavy sedation, prone positioning, and muscle blockers for a prolonged period which can increase the risk of acquiring secondary nosocomial infection, mainly ventilator-associated pneumonia with *Acinetobacter* spp. [49,50,51]. Nevertheless, an increased risk of *Acinetobacter baumannii* infections in non-critical COVID-19 patients is worrisome, given that the mortality associated with this etiologic agent has been reported to be as high as 85.7% [52].

The airway microbiota in healthy adults is colonized by members of *Streptococcus*, *Neisseria*, *Haemophilus*, and *Lachnospira* spp. [53]. A study by Yamamoto et al. on the lung microbiome reported differences between COVID-19 patients and healthy controls, with an increase in Staphylococcus, Streptococcus, and Enterobacterales species in the first group [54]. Sharov et al. analyzed 3382 cases of bacterial coinfections during the initial phase of the pandemic and identified *S. pneumoniae*, *S. aureus*, and *H. influenzae* as the most common agents associated with bacterial pneumonia [55]. Similarly, in this study, *S. pneumoniae* as part of the commensal human pathogens caused respiratory infections in 34.1% of patients, followed by methicillin-sensitive (21.6%) and methicillin-resistant *Staphylococcus aureus* (MRSA) (17.0%). We hypothesize that an imbalance in the respiratory microbiota such as in a SARS-CoV-2 infection can determine commensal organisms to act as pathogenic. ACE2, the receptor for SARS-CoV-2, is an interferon-stimulated gene and thus could be modulated by the respiratory microbiome [56]. In line with our findings, Merenstein et al. classifies *Staphylococcus aureus* as an emerging co-pathogen in COVID-19 [57]. As Romania ranks high amongst countries with a high antibiotic consumption per capita, MRSA rates continue to be extremely elevated, ranging from approximately 30% up to 70% in recent studies [58,59,60,61].

Among the etiologic agents responsible for fungal co-infections and superinfections, *Candida* spp. (53.4%) and *Aspergillus* spp. (41.1%) were the most frequently detected species. Opportunistic invasive fungal disease in the setting of severe respiratory viral illness is not novel, being well described in the context of severe influenza, parainfluenza, and respiratory syncytial virus infections [62,63]. Whilst COVID-19-associated pulmonary aspergillosis is a well-established clinical entity, reports of fungal coinfections due to yeasts and non-Aspergillus filamentous fungi have increased [64,65,66,67,68]. The incidence of COVID-19-associated candidiasis has ranged from 0.7% in Spain to 12.6% and 23.5% in the United Kingdom and China, respectively [69]. Although such a high rate of fungal co- and superinfections might come across as a potential overestimation, given the fact that our study population excluded critically ill patients, it can be explained in the context of poor oral hygiene, immunity dysregulation, and viral cytopathic effects on ductal epithelial cells [70,71]. According to a study in oral healthcare by Oancea et al., 80% of Romanians were found to have dental problems, emphasizing the need for cautious interpretation of fungal isolates from respiratory samples, especially in non-critically ill patients with a low pre-test probability of invasive fungal infections [72]. Lung epithelium damage exerted by SARS-CoV-2 facilitates *Candida* adherence to basement membrane causing subsequent invasive pulmonary candidiasis [68,73]. In this study, 17.6% of patients with fungal infections had a pre-existing lung disease.

Regarding the distribution of antimicrobial resistance among COVID-19 patients, 6.5% of samples and 4.5% of samples in the *Pathogenic bacteria* and commensal human pathogens groups, respectively, were resistant to more than five antimicrobials. This can be explained by the current context in which the combination of the fear of COVID-19 and the lack of adequate knowledge of the utility of antibiotics has a direct impact on over-the-counter access to antibiotics, especially in low- and middle-income countries such as Romania, with weak antibiotic control measures [69,74]. In Romania, the bacterial antimicrobial resistance level is far above the figures reported in Western Europe [75,76]. A study by Rawsan et al. has reported that 72% of COVID-19 patients attending hospitals have received antimicrobial agents, despite only 8% being co-infected by bacteria or fungi [77]. Langford et al. established antibiotic exposure as a significant risk factor for antimicrobial resistance in COVID-19 patients [6]. Khoshbakht et al., reported on a 30% rise in antibiotic resistance to Ceftriaxone for *Klebsiella pneumoniae* in Iran and Sulayyim et al. observed that the resistance of *K. pneumonia* to colistin increased during COVID-19, with a median of 21.1% [78,79]. At present, the national guideline recommends the use of empiric antibiotic therapy in patients with mild or moderate forms of the disease only if the bacterial infection is clinically suspected or in the presence of indicative biological or imaging evidence [80].

In this research, by performing a univariate analysis, we highlighted the significantly higher values of the inflammatory biomarkers, such as procalcitonin, IL-6, ESR, and CRP in patients with *Pathogenic bacteria* and commensal flora respiratory co-infections [14,81,82,83]. In line with previous studies, our data indicated that leukocytosis is an independent risk factor associated with bacterial infections [33,84].

While there may be a trend towards a higher hazard ratio for the fungal disease subgroup, the overlapping confidence intervals indicate that we cannot confidently assert a significant difference in hazard ratios between the culture-positive subgroups. However, data are contrasting in this regard, with reports indicating escalating mortality rates in COVID-19 patients with secondary infections, while other studies failed to demonstrate this mutual link [85,86,87]. Resistant Gram-negative bacteria continue to impose a significant economic burden on Romanian healthcare.

This study has limitations. By applying a strict definition of bacterial co-infection based on sputum samples taken within 48 h of admission, our study deliberately decreased sensitivity for bacterial co-infection overall, and excluded other types of pathogens. We eliminated culture results from bacterial species likely to represent cross-contamination. The validity of sputum culture is improved by strict case definition and adequate radiographic review, but in the absence of a gold standard, sputum diagnostics is often underexposed. Finally, COVID-19 vaccination status of the patients was not included in the statistical analysis due to the heterogeneity of the data (low vaccination rate or incomplete vaccination schedule recorded in the region and three available vaccines on the Romanian market).

## 5. Conclusions

Due to the controversy surrounding the utility of sputum samples, the diagnosis and management of individuals with fever and respiratory disease associated with COVID-19 remain challenging. As Romania has one of the highest rates of antibiotic use with evidence of self-medication and improper dosage use or therapy length associated with multidrug-resistant pathogens, there is a need for regionally tailored, cost-effective policies in infection control practices, especially in the intensive care setting. Antimicrobial stewardship interventions and carefully designed large individual prospective studies investigating the exact incidence and antimicrobial resistance patterns of COVID-19 secondary bacterial or fungal infections are of paramount importance in Romania, especially because the discovery of new therapeutic agents is not highly promising.

## Figures and Tables

**Table 1:** General characteristics of COVID-19 patients stratified by sputum culture results.

Variables	Positive Sputum			Negative Sputum (*n* = 243)	*p*-Value
	*Pathogenic bacteria* (*n* = 46)	Commensal Pathogens (*n* = 67)	Fungi (*n* = 51)		
Age					0.369
18–40 years	2 (4.3%)	4 (6.0%)	1 (2.0%)	14 (5.8%)	
40–65 years	19 (41.3%)	22 (32.8%)	25 (49.0%)	79 (32.5%)	
>65 years	25 (54.3%)	41 (61.2%)	25 (49.0%)	150 (61.7%)	
Age (mean ± SD)	62.1 ± 12.0	66.8 ± 13.7	64.4 ± 11.3	67.5 ± 14.8	0.073
Sex					0.900
Men	27 (58.7%)	35 (52.2%)	28 (54.9%)	129 (53.1%)	
Women	19 (41.3%)	32 (47.8%)	23 (45.1%)	114 (46.9%)	
Area of residence					0.649
Urban	24 (52.5%)	32 (56.9%)	29 (56.9%)	136 (56.0%)	
Rural	22 (47.8%)	35 (43.1%)	22 (43.1%)	107 (44.0%)	
BMI					0.539
Underweight (<18.5 kg/m^2^)	4 (8.7%)	4 (6.0%)	6 (11.8%)	14 (5.8%)	
Normal weight (18.5–25.0 kg/m^2^)	25 (54.3%)	40 (59.7%)	22 (43.1%)	130 (53.5%)	
Overweight (>25.0 kg/m^2^)	17 (37.0%)	23 (34.3%)	23 (45.1%)	99 (40.7%)	
Smoking status					0.195
No	12 (26.1%)	10 (14.9%)	9 (17.6%)	63 (25.9%)	
Yes	34 (73.9%)	57 (85.1%)	42 (82.4%)	180 (74.1%)	
Pre-existing lung disease					0.532
No	5 (10.9%)	6 (9.0%)	9 (17.6%)	33 (13.6%)	
Yes	41 (89.1%)	61 (91.0%)	42 (82.4%)	210 (86.4%)	
Comorbidities					0.166
0	3 (6.5%)	1 (1.5%)	2 (3.9%)	12 (4.9%)	
1–2	23 (50.0%)	35 (52.2%)	27 (52.9%)	155 (63.8%)	
≥3	20 (46.3%)	31 (46.3%)	22 (43.1%)	76 (31.3%)	

BMI—Body Mass Index; SD—Standard Deviation.

**Table 2:** Hospital admission features and testing results of COVID-19 patients, stratified by sputum culture results.

Variables	Positive Sputum			Negative Sputum (*n* = 243)	*p*-Value
	*Pathogenic bacteria* (*n* = 46)	Commensal Pathogens (*n* = 67)	Fungi (*n* = 51)		
Days from symptom onset until hospitalization (mean ± SD)	6.1 ± 1.4	5.9 ± 1.0	4.7 ± 1.5	6.8 ± 1.8	<0.001
Days from positive COVID-19 PCR test until hospitalization (mean ± SD)	4.2 ± 1.5	4.0 ± 1.4	4.2 ± 1.5	4.0 ± 1.4	0.359
Prior hospitalization					0.279
No	40 (87.0%)	59 (88.1%)	40 (78.4%)	215 (88.5%)	
Yes	6 (13.0%)	8 (11.9%)	11 (21.6%)	28 (11.5%)	
Time of sampling					0.520
Within 48 h from admission	28 (60.9%)	36 (53.7%)	31 (60.8%)	126 (51.9%)	
After 48 h from admission	18 (39.1%)	31 (46.3%)	20 (39.2%)	117 (48.1%)	
Multidrug resistance					0.283
Yes	42 (91.3%)	55 (82.1%)	41 (80.4%)	-	
No	4 (8.7%)	12 (17.9%)	10 (19.6%)	-	

SD—Standard Deviation.

**Table 3:** Distribution of antimicrobial resistance among COVID-19 patients stratified by sputum culture results.

Variables	Positive Sputum			*p*-Value
	*Pathogenic bacteria* (*n* = 46)	Commensal Pathogens (*n* = 67)	Fungi (*n* = 51)	
Distribution of antimicrobial resistance				
0 drug resistance	4 (8.7%)	12 (17.9%)	10 (19.6%)	0.283
1 drug resistance	7 (15.2%)	15 (22.4%)	11 (21.6%)	0.616
2 drug resistance	10 (21.7%)	18 (26.9%)	13 (25.5%)	0.822
3 drug resistance	11 (23.9%)	10 (14.9%)	14 (27.5%)	0.227
4 drug resistance	7 (15.2%)	6 (9.0%)	3 (5.8%)	0.289
5 drug resistance	4 (8.7%)	3 (4.5%)	0 (0.0%)	0.106
>5 drug resistance	3 (6.5%)	3 (4.5%)	0 (0.0%)	0.208

SD—Standard Deviation.

**Table 4:** Biological parameters of COVID-19 patients at admission, stratified by sputum culture results.

Variables		Positive Sputum			Negative Sputum (*n* = 243)	*p*-Value
	Normal Range	*Pathogenic bacteria* (*n* = 46)	Commensal Pathogens (*n* = 67)	Fungi (*n* = 51)		
RBC (millions/mm^3^)	4.35–5.65	6 (13.0%)	12 (17.9%)	11 (21.6%)	28 (11.5%)	0.207
PLT (thousands/mm^3^)	150–450	2 (4.3%)	4 (6.0%)	1 (2.0%)	14 (5.8%)	0.705
WBC (thousands/mm^3^)	4.5–11.0	34 (73.9%)	57 (85.1%)	29 (56.9%)	114 (46.9%)	<0.001
Lymphocytes (thousands/mm^3^)	1.0–4.8	11 (23.9%)	18 (26.9%)	27 (52.9%)	107 (44.0%)	0.002
Hb (g/dL)	13.0–17.0	7 (15.2%)	6 (9.0%)	10 (19.6%)	33 (13.6%)	0.413
Hematocrit (%)	36–48	10 (21.7%)	12 (17.9%)	8 (15.7%)	35 (14.4%)	0.620
Creatinine (µmol/L)	0.74–1.35	17 (37.0%)	23 (34.3%)	25 (49.0%)	97 (39.9%)	0.422
BUN (mmol/L)	2.1–8.5	12 (26.1%)	10 (14.9%)	9 (17.6%)	50 (20.6%)	0.497
GFR	>60	6 (13.0%)	10 (14.9%)	3 (5.8%)	12 (4.9%)	0.021
Fasting glucose (mg/dL)	72–125	23 (50.0%)	35 (52.2%)	27 (52.9%)	92 (37.9%)	0.048
ALT (U/L)	7–35	6 (13.0%)	8 (11.9%)	13 (25.5%)	26 (10.7%)	0.041
AST (U/L)	10–40	4 (8.7%)	9 (13.4%)	10 (19.6%)	22 (9.1%)	0.142
INR	1.1	5 (10.9%)	7 (10.4%)	7 (13.7%)	13 (5.3%)	0.125
Ferritin (ng/mL)	15–300	13 (28.3%)	44 (23.9%)	19 (37.3%)	62 (25.5%)	0.339
LDH (U/L)	100–300	16 (34.8%)	23 (34.3%)	20 (39.2%)	56 (23.0%)	0.037
Procalcitonin (ng/mL)	<0.5	28 (60.9%)	41 (61.2%)	12 (23.5%)	29 (11.9%)	<0.001
Lactate (mmol/L)	<1	18 (39.1%)	30 (44.8%)	35 (68.6%)	58 (23.9%)	<0.001
CRP (mg/L)	0–10	40 (87.0%)	59 (88.1%)	39 (76.5%)	124 (51.0%)	<0.001
IL-6 (pg/mL)	0–16	32 (69.6%)	37 (55.2%)	39 (76.5%)	133 (54.7%)	0.012
ESR (mm/h)	0–22	28 (60.9%)	32 (47.8%)	34 (66.7%)	95 (39.1%)	0.001
Fibrinogen (g/L)	2–4	31 (67.4%)	39 (58.2%)	30 (58.8%)	91 (37.4%)	<0.001
D-dimer (ng/mL)	<250	25 (54.3%)	46 (68.7%)	31 (60.8%)	94 (38.7%)	<0.001

RBC—Red Blood Cells; PLT—Platelets; WBC—White Blood Cells; Hb—Hemoglobin; BUN—Blood Urea Nitrogen; GFR—Glomerular filtration Rate; CRP—C-reactive Protein; IL—Interleukin; ESR—Erythrocyte Sedimentation Rate; LDH—Lactate dehydrogenase; INR—International Normalized Ratio.

**Table 5:** Outcomes of COVID-19 patients stratified by sputum culture results.

Variables	Positive Sputum			Negative Sputum (*n* = 243)	*p*-Value
	*Pathogenic bacteria* (*n* = 46)	Commensal Pathogens (*n* = 67)	Fungi (*n* = 51)		
Severe complications					0.028
Yes	16 (34.8%)	23 (34.3%)	20 (39.2%)	55 (22.6%)	
No	30 (65.2%)	44 (65.7%)	31 (60.8%)	187 (77.4%)	
COVID-19 severity					0.122
Mild	5 (10.9%)	7 (10.4%)	4 (7.8%)	28 (11.5%)	
Moderate	18 (39.1%)	31 (46.3%)	15 (29.4%)	119 (49.0%)	
Severe	23 (50.0%)	29 (43.3%)	32 (62.7%)	96 (39.5%)	
Oxygen saturation at admission					0.040
≤98%	7 (15.2%)	9 (13.4%)	5 (9.8%)	38 (15.6%)	
92–97%	16 (34.8%)	27 (40.3%)	13 (25.5%)	112 (46.1%)	
<92%	23 (50.0%)	31 (46.3%)	33 (64.7%)	93 (38.3%)	
Oxygen supplementation at admission					<0.001
Yes	34 (73.9%)	46 (68.7%)	41 (80.4%)	117 (48.1%)	
No	12 (26.1%)	21 (31.3%)	10 (19.6%)	126 (51.9%)	
Oxygen flow rate (L/min)	12.8 ± 4.6	12.0 ± 5.1	12.8 ± 4.6	12.0 ± 5.1	0.120
Outcomes					0.118
ICU admission	15 (32.6%)	21 (31.3%)	18 (35.3%)	42 (17.3%)	0.003
Days in the ICU (mean ± SD)	7.9 ± 2.2	7.3 ± 1.8	9.5 ± 2.4	6.6 ± 1.3	<0.001
Mortality	13 (28.2%)	15 (22.4%)	14 (27.5%)	34 (14.0%)	0.023
Days from admission until death (mean ± SD)	8.8 ± 2.0	8.5 ± 1.5	10.2 ± 2.4	8.3 ± 1.1	<0.001
Days of hospitalization among survivors (mean ± SD)	14.6 ± 5.2	13.8 ± 4.5	17.2 ± 6.0	12.0 ± 4.3	<0.001

ICU—Intensive Care Unit.

## Data Availability

Data available on request.
